# Evaluating the impact of Archway: a personalized program for 1st year student success and mental health and wellbeing

**DOI:** 10.1186/s12889-020-10057-0

**Published:** 2021-01-06

**Authors:** Matthew Y. W. Kwan, Denver Brown, James MacKillop, Sean Beaudette, Sean Van Koughnett, Catharine Munn

**Affiliations:** 1 Department of Child and Youth Studies, Brock University, 812 Sir Isaac Brock Way, St. Catharines, Ontario L2S 3A1 Canada; 2 Department of Family Medicine, McMaster University, Hamilton, Ontario L8S 4L8 Canada; 3 Department of Psychiatry and Behavioural Neurosciences, McMaster University, Hamilton, Ontario L8S 4L8 Canada; 4 Housing and Conference Services, McMaster University, Hamilton, Ontario L8S 4L8 Canada; 5 Student Affairs, McMaster University, Hamilton, Ontario L8S 4L8 Canada

**Keywords:** Transition, Emerging adulthood, Support program, Mental health, Wellbeing

## Abstract

**Background:**

First-year students entering postsecondary education must navigate a new and complex academic and social environment. Research indicates that this transition and developmental period can be challenging and stressful – academically, emotionally and socially – and that mental health and wellbeing can be compromised. Additionally, mental health disorders can also compromise students’ ability to successfully navigate this transition. In the COVID-19 pandemic, the incoming 2020 cohort of first-year students face heightened and new challenges. Most will have spent the conclusion of high school learning virtually, in quarantine, in an uncertain and difficult time, and are then experiencing their first year of university while living, learning and socializing off-campus, virtually and remotely. In response to COVID-19 and with an appreciation of the considerable stresses students face generally and particularly in 2020-21, and the potential effects on mental health and wellbeing, McMaster University, a mid-sized research intensive university with approximately 30,000 students, has developed an innovative program to support students, called Archway. This initiative has been developed to help to prevent and to intervene early to address common transitional issues students experience that can influence mental health and wellbeing, with the ultimate goals of increasing student connectedness, supports, and retention.

**Methods:**

The current study will use a mixed-method design to evaluate Archway and gain a better understanding of the transition into first-year postsecondary for students who engage and participate in Archway at various levels. The study will not only help to determine the effect of this program for students during COVID-19, but it will help us to better understand the challenges of this transition more broadly.

**Discussion:**

Findings have the potential to inform future efforts to support students and protect their mental health and wellbeing through the use of virtual and remote platforms and mechanisms that meet their increasingly diverse needs and circumstances.

## Background

The transition into postsecondary education is particularly challenging for many as it often coincides with living away from home for the first time, changes in relationships, and a loss of social support systems [1, 2]. Recently, results from a longitudinal study of 5532 American undergraduate students found a sharp rise in students’ psychological distress over the first semester, which remained elevated throughout the first few years at university [3]. While most emerging adults are generally able to navigate the transition such that they continue school beyond first-year university, there is evidence that many undergraduate university students nonetheless report high levels of stress, anxiety and depression, with 26% of students reporting feeling so depressed, and 43% of students reporting feeling so overwhelmed by anxiety in the past month, that it was difficult to function [4]. These challenges are often further exacerbated for students from minority or marginalized groups, including racialized students, Indigenous students, first generation university students, international students, those identifying as gender non-conforming and LGBTQ2S+, students living in poverty, and students exposed to trauma, among others [5].

The pathways leading to the development of mental health disorders or wellbeing are complex and multi-factorial; however, psychosocial stress is central to them [6, 7]. Academic difficulties and failure are also associated with poor mental health among post-secondary students, with evidence suggesting that each of these is both a predictor and a consequence of mental health challenges [8–10]. Additionally, stress, distress, mental health and substance use symptoms and disorders are associated with significant impairments in cognitive, social, and emotional functioning, which increases the risk of drop-out, lower educational attainment, and suicidal behaviour [11–13]. Loneliness is another significant and growing social factor which has been studied among young adults across genders and socioeconomic status. Several studies have shown perceptions of loneliness to be associated with negative mental health and health outcomes, poorer life satisfaction, and more negative coping strategies [14–16]. A lack of social bonds and social exclusion, related to loneliness, have also been shown to contribute to anxiety; and conversely, possessing a sense of belonging is strongly associated with happiness and subjective wellbeing [17–19]. Studies of students also demonstrate that having a sense of belonging is associated with reduced loneliness, social anxiety, and depression, suggesting that it may be a protective factor for mental health among post-secondary students [20–22]. Overall, interventions that assist and promote a sense of belonging have demonstrated mental health and academic benefits [23].

Prior to the pandemic, post-secondary student mental health and wellbeing was a growing concern, but the COVID-19 pandemic appears to have exacerbated the problem. Globally, since the World Health Organization declared a global health crisis in March 2020, post-secondary students have reported increased levels of stress, anxiety and depressive symptoms [24–29]. Early research indicates that the pandemic may be taking a greater toll on the mental health of first-year students [28] and students from minority or marginalized groups [30, 31]. The declines in student mental health have coincided with increased feelings of loneliness and social isolation, to some degree attributable to the public health quarantine measures implemented to stop the spread of the virus (e.g., stay at home orders, social distancing) [25, 32]. Increases in academic-related concerns to adapting to online learning have also been found to underly the significant uptick in mental health problems [26, 28]. Unfortunately, many students are struggling to cope with these challenges related to the current situation [28]. These issues have sparked several calls for postsecondary institutions to develop innovative procedures and programming to meet students’ needs during the pandemic [33, 34].

### The Archway program: Theoretical Foundation

McMaster University decided early Spring that students would not be on campus for the upcoming 2020–21 academic year in order to protect student health and safety during COVID-19. Given the recognition of the potential academic, social and emotional consequences, the institution moved quickly to develop a program to address the resulting and inherent challenges for students, and to address gaps in their experiences and supports. The program, titled Archway (https://archway.mcmaster.ca/), has been designed to remotely provide students with the benefits of the on-campus academic and social experience during the pandemic. There is an understanding that differing needs exist for each incoming student, thus Archway is intended to provide an overarching support structure for all students, while remaining flexible enough that individual students can receive tailored support.

The provision of support through Archway is guided by several prominent theoretical frameworks. First, according to Schlossberg’s Transition Theory [35], it must be recognized that students will face both anticipated and unanticipated transitional issues as they navigate major changes in their lives. Second, Sanford’s Theory of Challenge and Support [36] posits that student development is maximized when challenges are met with supports that can sufficiently tolerate the stress of the challenge itself. While the more obvious barrier is not having enough support, the reality is that too much support can also be limiting as students struggle to become independent to define their own beliefs, identity, and relationships. Third, Baumeister and Leary’s Need to Belong Theory [37], and evidence stemming from this work, argues and demonstrates that human beings are motivated, or even “wired”, to establish sufficient stable and positive interpersonal relationships. There is strong empirical evidence that a sense of belonging is critical in higher education, as it has been found to be associated with a variety of positive outcomes including academic retention and overall health and wellbeing [18]. Similarly, development of a social identity has also been found to form a buffer against the stresses of life transitions [38]. Archway is a program which intends to meet the unique needs of first-year students by helping them to engage and cultivate a sense of belonging to the student and university communities, and by providing each student with the necessary support, information and services to help them with the transition to postsecondary, with particular attention to their mental health and wellbeing during COVID-19. Referrals to specialized forms of peer support (e.g., access for students with disabilities, Women and Gender Equity Network), and professional support (e.g., Student Accessibility Services, Student Success Centre) can be considered as appropriate.

### The Archway program

The Archway program will be made available to all 7000+ incoming 2020 first-year students at McMaster University with an understanding that not every student will enroll, engage, and/or participate at the same level.

#### Virtual infrastructure

Microsoft Teams will be the main platform used for the program, enabling live meetings to be organized and facilitated, and relevant information, links and documents to be housed on a single site for ease of access during the transition. For example, there will be direct links to the Registrar’s Office (e.g., to access documentation about course enrolment and registration); the Student Wellness Centre (e.g., for access to health-related infographics or information about remote access to care); Faculties and programs (with weekly bulletins for different programs on campus); McMaster Students Union (e.g., services, club information); and McMaster Athletics and Recreation (e.g., free online programming to stay active). Importantly, Archway will have the reach of all aspects of campus. Microsoft Teams was selected as an intuitive, accessible, and secure communication platform, whereby students will also have the opportunity to engage with other students, and within different Archway Communities within Teams. Importantly, there will be the ability to track participation and engagement using Teams. Other virtual platforms, such as Twitch for eGaming will be used to supplement community activities where appropriate.

#### Archway Coaches & Mentors

*Archway Coaches* are full-time Student Affairs employees, that is, staff who are well-positioned to proactively support students in navigating and accessing university services and to supervise Archway Mentors. Each will be trained to make personal referrals to resources on campus such as Academic Advisors, the Student Wellness Centre, and the Office of Equity and Inclusion. *Archway Mentors* are paid upper-year undergraduate students who will provide additional support from a student perspective, having recently transitioned into university themselves. Each student will be assigned an Archway Coach and Mentor and placed in an Archway Community with approximately 35 students, grouped by identified interests. For example, communities will be developed based on self-identified interests such as sports, wellness, gaming, cooking, and coding. The aim of these small virtual communities is to promote a sense of belonging and social identity among first-year students within the groups. Archway Coaches and Mentors will be integrated within these communities, acting as navigators to connect groups or individuals with group activities that will help build cohesion within their Archway Community, and supports as needed. Coaches and Mentors will also organize and guide two sessions per month, delivered and available both synchronously and asynchronously to maximize reach. For example, Coaches will hold sessions on topics such as study tips and coping with deadlines. The Mentor-led sessions will be customizable and focused on making social connections by responding to student interest within each Archway community in order to maximize engagement. For example, activities could include viewing parties, speed-meeting, drop-in study groups, cooking classes/challenges, book clubs, among others. Mentors will be provided with facilitation guides and scripts, which, in coordination with Coaches and the Office of Student Affairs, will enable them to lead activities and conversations aligned with the transitional needs of students. Beyond group-based activities, Archway Coaches and Mentors will be available to meet individual transitional needs by drawing on the 4 S’s outlined in Schlossberg’s Transition Theory: situation, self, support, and strategies [39]. The broader vision for Archway is to help students through all phases of their transition into McMaster University, from the time they ‘move into’ their virtual campus until they enter their second year of studies, engaging and connecting them, and proactively and reactively supporting and informing them.

Using a Population Health Model [40], Archway Coaches and Mentors will contact each student to assess their needs and make support plans appropriate to the individual. For example, students who express a need for more ongoing support would be identified as “High Need”, requiring more frequent meetings with their Archway Coach, Mentors, and connections to other campus supports. The focus for “Low Need” students will be on enhancing connection to the student community, with fewer one-on-one check-ins with their Archway Coach and more encouragement to participate in community-based opportunities. “Moderate Need” students are those who may not know their needs or be recognized initially as needing support, but are identified by mentors over time as having higher needs. Enrollment and engagement in Archway will be tracked through Microsoft Teams. The purpose of this study will be to examine the effectiveness of the Archway program being delivered to a cohort of first-year university students.

## Methods

### Research design

The study will use a mixed-method design, with two studies to evaluate effectiveness for the Archway program. **Study 1** will be a longitudinal cohort study to determine if participants enrolled in Archway experience sustained or improved academic retention, social connectedness, mental health and well-being and use of supports when compared to first-year students who do not enroll into Archway. **Study 2** is a qualitative study that will investigate the experiences, strengths and limitations of Archway from the student perspective, while also exploring the reasons behind low engagement or non-enrollment.

#### Study 1

The Canadian Campus Wellbeing Survey (CCWS) will be administered at the beginning and end of the 2020/2021 academic year as the primary evaluation tool (*see* www.ccws-becc.ca). The CCWS was recently developed as a platform to help monitor student wellbeing and support implementation/evaluation of interventions to promote student wellbeing at the post-secondary level [41]. Importantly, the CCWS was recently piloted (March 2020) to half of McMaster’s undergraduate student population and found to be a feasible and effective assessment tool. Specifically, the data collection system will be administered through the Office of Institutional Research and Analysis (IRA), whereby individual CCWS responses will be linked with critical student-based information (e.g., program of study, enrollment, etc.). All first-year students will be invited to complete the baseline assessment in September 2020; and baseline respondents will be invited to complete the follow-up assessment in March 2021. To encourage participation, participants will be entered into a draw for one of 100 ($100 CAD) gift cards and a grand prize for one-semester of tuition ($5000 CAD value). The goal will be to achieve a 50% response rate with a modest 20% attrition rate, representing a high response rate for larger higher education surveys [42].

### Measures (within the Canadian campus wellbeing survey)

The CCWS tool consists of well-validated measures across eight domains: *mental health assets*, *mental health deficits*, *campus climate*, *health service utilization*, *physical health*, *health behaviors*, *substance u*s*e*, and *academic achievement*. Our primary measures of interest within the CCWS include the Warwick-Edinburgh Mental Wellbeing Scale (WEMWBS) [43]; Brief Resiliency Scale [44]; Kessler Psychological Distress Scale (K-10) [45]; Generalized Anxiety Disorder Scale (GAD-7) [46]; Sense of Belonging Scale [47]; Perceptions of Social Isolation [48]; and Perceptions of Academic Support [47]. Secondary measures of interest within the CCWS includes responses to questions from the Canadian Student Tobacco, Alcohol, and Drugs Survey [49]. All measures are to be assessed at baseline and follow-up. To determine rates of continuation and drop-out, the Office of the IRA will also be able to determine student retention through second semester registration data.

### Data analyses

To answer our primary research question on program effectiveness, we will use mixed effects modeling to examine changes in each of our primary measures of interest (i.e., mental wellbeing, resiliency, psychological distress, sense of belonging, social isolation, and academic support) between baseline and follow-up, testing for differences based on students’ enrollment in Archway (i.e., enrolled vs not enrolled) and levels of engagement with Archway among those who enroll (i.e., high, moderate, or low users). Covariates hypothesized to influence the primary outcomes (e.g., gender, socioeconomic status, ethnicity or BIPOC status, geographic location) will be included in all analyses. Secondarily, because McMaster administered the CCWS to a cohort of first-year students in March 2020 (pre-COVID), exploratory analyses will be conducted to examine if there are significant differences in mean-level outcomes between the different cohorts (i.e., pre- and during COVID-19). Additionally, the large sample size will enable person-centred analysis (e.g., latent profile analysis) to identify subgroups of the student population that may require focused attention based on differences in mental health assets and deficits.

#### Study 2

Virtual interviews will be conducted with first-year students to capture an in-depth understanding of students’ thoughts, experiences and insights related to participation in Archway. Specifically, the aim is to recruit high (weekly), moderate (monthly), and low (occasional) Archway users, as well as students who did not enroll into Archway to begin with. It is estimated that 12-15 students in each specific group will be interviewed, or until data saturation is reached. Purposive sampling will be used to ensure diversity in gender, socioeconomic status, ethnicity and geographic location (i.e., time zone) within the groups.

### Interview guide

The interview is designed to elicit feedback on Archway as it relates to Study 1 outcomes with an intensive focus on mental health and wellbeing, as well as on-campus climate (i.e., sense of belonging, feelings of social isolation, perceived academic support). Participants will be encouraged to share their positive and negative experiences as they related to these specific domains. Lastly, their insights and ideas regarding how Archway can be improved to better support student needs will be elicited. Interviews with non-enrollees will be conducted primarily to understand barriers for participation in Archway, to solicit their feedback to better engage students, and to identify the salient domains (e.g., mental wellbeing, resiliency, psychological distress, sense of belonging, social isolation, and academic support) in which these students might have benefited from intervention efforts.

### Data analyses

We will use an inductive thematic analysis approach to identify common themes within the data. Thematic analysis will be consistent with Braun and Clarke’s six phase approach [50]: (1) familiarization with the data, (2) generating initial codes, (3) searching for themes, (4) reviewing themes, (5) defining and naming themes, and (6) producing the report. The identified themes will be the strengths and limitations of Archway in relations to: (a) student health and wellbeing and (b) the school experience.

## Discussion

The Archway program was developed specifically in response to the COVID-19 pandemic, however the importance of better supporting students’ transition to post-secondary education generally, with particular attention to student mental health and wellbeing, has been recognized. McMaster University has been a national leader in this regard: (a) heavily investing in the development of a comprehensive mental health strategy; (b) being among the first institutions to form an Okanagan Charter Committee (https://okanagan.mcmaster.ca), implementing strategies to target the health and wellbeing for all on campus; and (c) creating the innovative Archway to support first-year students’ transition during COVID-19. The inclusion of enrollees and non-enrollees enables us to conduct a natural experiment to determine the effectiveness of Archway to support student success and mental health and wellbeing. These findings can directly inform future interventions designed to support first-year students in their transition, even after this pandemic has ended. Creating alternative virtual channels and platforms may help post-secondary institutions to better engage and support diverse populations of students in novel ways in the future, of particular importance given the mounting mental health concerns of students and growing demands for remote learning opportunities.

## Data Availability

Not applicable.
